# Exploring the Interplay between Complex Post-Traumatic Stress Disorder and Obsessive–Compulsive Disorder Severity: Implications for Clinical Practice

**DOI:** 10.3390/medicina60030408

**Published:** 2024-02-28

**Authors:** Martina D’Angelo, Marta Valenza, Anna Maria Iazzolino, Grazia Longobardi, Valeria Di Stefano, Giulia Visalli, Luca Steardo, Caterina Scuderi, Mirko Manchia, Luca Steardo

**Affiliations:** 1 Psychiatry Unit, Department of Health Sciences, University of Catanzaro Magna Graecia, 88100 Catanzaro, Italy; martina.dangelo001@studenti.unicz.it (M.D.); iazzolinoanna@gmail.com (A.M.I.); grazia.longobardi@studenti.unicz.it (G.L.); valeria.distefano@studenti.unicz.it (V.D.S.); giulia.visalli@studenti.unicz.it (G.V.); steardo@unicz.it (L.S.J.); 2 Department of Physiology and Pharmacology “Vittorio Erspamer”, Sapienza University of Rome, 00185 Rome, Italy; marta.valenza@uniroma1.it (M.V.); luca.steardo@uniroma1.it (L.S.); caterina.scuderi@uniroma1.it (C.S.); 3 Department of Clinical Psychology, University Giustino Fortunato, 82100 Benevento, Italy; 4 Unit of Psychiatry, Department of Medical Sciences and Public Health, University of Cagliari, 09124 Cagliari, Italy; 5 Unit of Clinical Psychiatry, University Hospital Agency of Cagliari, 09124 Cagliari, Italy; 6 Department of Pharmacology, Dalhousie University, Halifax, NS B3H 4R2, Canada

**Keywords:** observational study, trauma, stress, clinical course

## Abstract

*Background and Objectives*: Traumatic events adversely affect the clinical course of obsessive–compulsive disorder (OCD). Our study explores the correlation between prolonged interpersonal trauma and the severity of symptoms related to OCD and anxiety disorders. *Materials and Methods*: The study follows a cross-sectional and observational design, employing the International Trauma Questionnaire (ITQ) to examine areas linked to interpersonal trauma, the Hamilton Anxiety Rating Scale (HAM-A), and the Yale–Brown Obsessive–Compulsive Scale (Y-BOCS) to assess anxious and obsessive–compulsive symptoms, respectively. Descriptive analysis, analysis of variance (ANOVA), and logistic regression analyses were conducted. *Results*: We recruited 107 OCD-diagnosed patients, categorizing them into subgroups based on the presence or absence of complex post-traumatic stress disorder (cPTSD). The ANOVA revealed statistically significant differences between the two groups in the onset age of OCD (*p* = 0.083), psychiatric familial history (*p* = 0.023), HAM-A, and Y-BOCS (*p* < 0.0001). Logistic regression indicated a statistically significant association between the presence of cPTSD and Y-BOCS scores (*p* < 0.0001). *Conclusions*: The coexistence of cPTSD in OCD exacerbates obsessive–compulsive symptoms and increases the burden of anxiety. Further advancements in this field are crucial for mitigating the impact of early trauma on the trajectory of OCD and associated anxious symptoms.

## 1. Introduction

Recently, studies on psychological trauma have witnessed significant growth [1]. Between 13 and 30% of individuals report having encountered traumatic experiences during childhood [2], which have enduring ramifications on their physical and mental well-being [3]. Consequently, chronic exposure to childhood traumatic events is closely associated with an elevated likelihood of developing Complex Post-traumatic Stress Disorder (cPTSD) [3]. Indeed, cPTSD has gained recognition as a novel diagnosis in the Eleventh International Classification of Diseases (ICD-11) [4,5]. The proposition of a repeated trauma-related disorder, as articulated by Herman, underscores the potentially profound impact of prolonged traumatic stressors, particularly on self-organization, with a focus on the affective and relational domains [6]. cPTSD manifests as a severe mental disorder emerging in response to traumatic life events, encompassing a constellation of symptoms stemming from cumulative interpersonal traumas experienced throughout developmental stages [7]. Notably, cPTSD is characterized by three primary clusters of post-traumatic symptoms alongside chronic, pervasive disturbances in emotion regulation, identity, and relationships [7]. A burgeoning body of literature demonstrates how traumas might serve as causal factors for a spectrum of outcomes, including emotional dysregulation, behavioral dysfunction, challenges in interpersonal relationships, and dissociative symptoms in adulthood [8]. cPTSD often ensues from unstable and distressing environmental contexts that negatively impact a child’s self-regulation skills, emotional equilibrium, psychological well-being, and interpersonal bonds [9]. Notably, particular attention has been devoted to the manifestation of self-organization disorders (DSO) observed within this condition, encompassing symptomatology spanning affective dysregulation, negative self-concept, and disrupted relationships [4]. Dysregulation, in this scenario, is a state of distress or impairment associated with the inability to tolerate aspects of one’s internal experience, including emotions, thoughts, bodily sensations, and physiological arousal. Activated by an actual or perceived threat, traumatic reminders, and other environmental or internal cues that trigger a person’s survival-driven alarm response. This can take a variety of forms, including heightened reactivity or shutdown. Strengthening one’s capacity for healthy self-regulation is a primary focus of complex trauma intervention with children and adults, leading to a higher psychopathological burden [10]. Moreover, alterations in consciousness and the emergence of dissociative symptoms disorganize individuals’ functioning across various levels, encompassing the biological, physiological, relational, and behavioral domains [11]. Individuals afflicted with cPTSD typically undergo prolonged or recurrent exposures to interpersonal trauma, such as childhood abuse or domestic violence [12,13,14]. The lifetime prevalence of Post-Traumatic Stress Disorder (PTSD) varies from 3 to 9% within the adult population, contingent upon the nature and frequency of traumas experienced. Meanwhile, the prevalence of cPTSD ranges from 1 to 8% in the general population, escalating to 50% within mental health settings [15]. 

Notably, childhood trauma has been associated with the onset of obsessive–compulsive disorder (OCD). Scientific literature posits that predisposing factors for this disorder, such as genetic vulnerability and typical OCD characteristics, interact with prolonged exposure to stressful and traumatic events during a child’s formative years [16,17,18,19,20]. Psychological distress often manifests as intrusive thoughts (flashbacks, nightmares) related to the traumatic experience, occasionally leading to anxiety, fear, aggression, anger, or depressive symptoms [21,22]. Recent studies suggest that OCD can emerge as a response to profoundly distressing events, with individuals exposed to trauma being more susceptible to developing OCD [23]. Childhood trauma exerts a profound impact on the development, progression, and severity of obsessive–compulsive symptoms, encompassing diverse clinical presentations [24]. Furthermore, previous trauma exposure among individuals with OCD has been correlated with greater functional impairment [25,26].

Childhood maltreatment, encompassing emotional, physical, and sexual abuse, as well as emotional and physical neglect, amplifies the vulnerability to physical ailments [27] and mental health conditions in adulthood [28], determining significant costs for both individuals and society [29]. Previous investigations into the impact of childhood maltreatment on psychopathology have predominantly centered on mental health disorders such as PTSD, mood disorders, personality disorders, and substance use disorders [30]. While preliminary evidence suggests a link between child abuse and neglect and the onset and persistence of OCD, the available data are limited and conflicting. While many prior studies have reported higher rates of childhood maltreatment among individuals with OCD compared to control groups [31,32,33,34,35], other well-powered studies have not found elevated prevalence rates of any form of childhood trauma in OCD patients [36,37]. Research indicates that exposure to emotional, physical, and sexual abuse correlates with heightened symptoms of OCD overall, implying that various traumatic experiences contribute to the severity of symptoms. Studies also reveal that exposure to childhood trauma is linked to increased symptoms across specific domains of OCD, including contamination, responsibility for harm, unacceptable thoughts, symmetry, aggression, sexual and religious obsessions, as well as ritualistic compulsions [38]. These individual differences and the overarching impact of childhood trauma on OCD severity underscore the importance of examining different subtypes of childhood trauma and OCD symptoms rather than solely focusing on total scores. Previous findings align with this line of research, suggesting that different subtypes of childhood trauma may be associated with distinct OCD symptoms such as obsessions or compulsions [39,40].

In addition, studies exploring the relationship between OCD severity and different subtypes of childhood trauma across various clinical presentations beyond OCD also highlight such association. Specifically, physical abuse, emotional abuse, and physical neglect are commonly associated with heightened OCD severity. The prevalence of OCD symptoms extends beyond OCD itself, suggesting the importance of investigating OCD symptoms across a spectrum of psychiatric disorders. Indeed, the co-occurrence of childhood trauma and OCD symptoms may heighten the risk of developing psychiatric disorders overall. For example, Barzilay et al. (2019) discovered in a community sample that individuals reporting sub-threshold OCD symptoms and exposure to stressful life events also reported elevated rates of depression, suicidal ideation, and psychosis [41]. Furthermore, epidemiological studies have identified numerous potential risk factors for OCD, such as age, gender, employment, and socioeconomic status, which are not exclusive to OCD [42]. This underscores the value of adopting a transdiagnostic approach to mental health. Similarly, childhood exposure to trauma has been linked to unfavorable outcomes across a range of psychiatric disorders, including functional impairment, a more severe illness trajectory, and an increased risk of chronic fatigue or pain [33]. Traumatic experiences, particularly those occurring during childhood, represent the most extensively studied etiological factor in the development of dissociation [43]. In clinical contexts, dissociation constitutes a core symptom across various disorders, including obsessive–compulsive disorder and Complex Post-traumatic Stress Disorder [44]. Dissociative phenomena serve as defense mechanisms against external traumatic experiences, with obsessions and compulsions often arising as responses to thoughts that intensify dissociation [45]. Psychological trauma is recognized as a risk factor in the development of dissociation, with numerous empirical studies substantiating the association between dissociation and trauma, especially severe childhood maltreatment [46,47,48]. Despite the numerous studies conducted on the topic and the significant clinical implications it may have, to date, no study has been conducted to assess the impact of cPTSD on OCD. In this context, this study aims to ascertain whether the presence of cPTSD is linked to heightened obsessive–compulsive symptoms and related anxiety symptomatology. Given that cPTSD represents a substantial domain of symptoms within the spectrum of psychiatric disorders, unraveling this correlation might assist clinicians in optimizing treatment strategies for OCD in the context of this comorbidity.

## 2. Materials and Methods

### 2.1. Clinical Assessment

The present study was designed as an observational cross-sectional investigation conducted in a clinical setting. The study recruited consecutive patients with OCD at the Psychiatry Unit of the “Magna Graecia” University of Catanzaro from January 2021 to April 2022. All participants were thoroughly informed about the research protocol’s objectives, data protection, privacy, and anonymity maintenance. Their participation was voluntary, contingent on providing formal consent in writing after a comprehensive explanation of the study’s aims and design. The study adhered to the latest version of the Declaration of Helsinki and gained approval from the Ethics Committee of the University of Catanzaro on 25 November 2020 (Ethics Committee Approval No. 307/2020).

Inclusion criteria were the following: (1) Patients diagnosed with OCD based on DSM-5 criteria, as determined by clinical interviews and psychometric assessments (Structured Clinical Interview for DSM-5—Clinical Version, SCID-5-CV). (2) Patients aged between 18 and 75 years. The exclusion criteria were as follows: (1) Patient refusal to participate. (2) Presence of significant neurological or psychiatric disorders (e.g., epilepsy, cognitive disability, dementia, Parkinson’s, genetic syndromes with psychiatric symptoms, and substance abuse). (3) Any condition hindering comprehensive assessment, such as language barriers or severe cognitive disabilities (e.g., dyslexia). Participants underwent a series of clinical and psychopathological evaluations conducted during outpatient clinical visits. Psychometric rating scales and sociodemographic data collection were carried out by researchers, medical specialists in training, and PhD students. OCD diagnoses were established according to the DSM-5 criteria, utilizing the SCID-5-CV [49,50]. Each enrolled patient underwent a semi-structured clinical interview to gather clinical and anamnestic information. Sociodemographic and clinical data were collected using a customized medical history questionnaire developed within our department. 

Patients clinical and sociodemographic characteristics, including gender, age at study entry, employment status, educational level, family history of psychiatric illnesses, type of onset, the pattern of illness course, treatments, suicidal ideation, previous psychiatric hospitalizations, and the presence of trauma were recorded according to an ad hoc schedule. In the schedule we developed for this study, there were a series of questions aimed at investigating the presence of trauma, including the following categories: natural disasters; serious work-related accidents or potentially life-threatening injuries (confirmed through medical reports); physical abuse; sexual abuse; and a subsection labeled “other” where we described the particularly stressful event witnessed. 

The assessment instruments employed included the Yale–Brown Obsessive–Compulsive Scale (Y-BOCS), the International Trauma Questionnaire (ITQ), and the Hamilton Anxiety Scale (HAM-A). Y-BOCS, renowned as the gold standard for assessing obsessive–compulsive symptoms, comprises a comprehensive symptom checklist categorized into various groups. This scale, which assesses obsessions distinct from compulsions, precisely gauges the intensity of symptoms related to obsessive–compulsive disorder without exhibiting prejudice toward or against the content of the obsessions or compulsions. Additionally, it incorporates a 10-item severity scale evaluating time, interference, discomfort, resistance, and symptom control [50,51]. Each item is rated on a scale of 0 to 4, yielding a total score ranging from 0 to 40, with higher scores indicating more severe OCD symptoms [52]. The ITQ was used to assess the trauma experienced and to diagnose cPTSD according to the ICD-11 guidelines. The questionnaire consists of eighteen items measuring the main symptoms of PTSD and DSO. The items can be answered on a 5-point Likert scale from 0 (not at all) to 4 (very strongly). The maximum score for PTSD and/or DSO is, therefore, 24 (range 0–24), while the maximum score for cPTSD is 48 (range 0 to 48). All items can only be considered present if they have a value ≥ 2 on the Likert scale. Diagnosis of cPTSD requires the endorsement of one of two symptoms from each of the three PTSD symptoms clusters (re-experiencing, avoidance, and sense of current threat) and one of two symptoms from each of the three Disturbances in Self-Organization (DSO) clusters: (1) affective dysregulation, (2) negative self-concept, and (3) disturbances in relationships. Functional impairment is present when at least one cluster of functional impairment is associated with PTSD symptoms and one with DSO symptoms. In principle, a person can only receive one of the two diagnoses, namely PTSD or cPTSD. Furthermore, cPTSD is characterized by the predominance of symptoms of disturbances in self-organization and is defined as that set of symptoms resulting from cumulative interpersonal traumas experienced during development: stories of abuse and repeated maltreatment in the family, severe neglect and abandonment, conditions of torture or imprisonment, wars, and forced migrations. When a person cannot escape the threat for a long time or when the threat occurs within the family upon which one must continue to depend for survival, the mind deploys more intense strategies to overcome the paradox and the pervasive state of fear. This is referred to as chronic traumatization rather than a single traumatic event. This questionnaire aligns with the principles of ICD-11, providing both categorical diagnostic scores and dimensional severity scores [53,54]. HAM-A, a 14-item rating scale, assesses the severity of anxiety symptoms, encompassing both psychological and somatic manifestations. It is one of the most widely used tests both in scientific research and in clinical practice and measures both psychic anxiety (mental agitation and psychological distress) and somatic anxiety (physical complaints related to anxiety). Each item is scored from 0 (absent) to 4 (severe), resulting in a total score ranging from 0 to 56. Severity levels are categorized as mild (<17), mild to moderate (18–24), and moderate to severe (25/30) [55,56,57].

### 2.2. Statistical Analysis

Data for all variables were entered into an electronic dataset. Descriptive statistical analyses were conducted to assess the distributional characteristics of sociodemographic and clinical variables within the sample. Continuous variables were presented as means with standard deviations (SD), while categorical variables were expressed as frequencies and percentages (%). The sample was categorized into two groups: those with a history of cPTSD (cPTSD+) and those without (cPTSD−). Analysis of variance (ANOVA) was employed to compare variances among the means of different groups, considering *p* < 0.005 as statistically significant. Regression analysis was performed to investigate the association between cPTSD and the Y-BOCS scale score. Statistical analyses were carried out using the Statistical Package for the Social Sciences version 26 (SPSS, Chicago, IL, USA).

## 3. Results

### Socio-Demographic Data

The final cohort for our investigation comprised 107 individuals diagnosed with OCD, with 50 of them (46.7%) additionally diagnosed with comorbid cPTSD. The sociodemographic profile of the sample exhibited homogeneity, with 54 (50.5%) male participants. 84 subjects (78.5%) had diploma. Patients with a co-occurrence of OCD and cPTSD exhibited a familial psychiatric history prevalence of 69.2%. Within this segmented sample, the average age among OCD patients was 45.75 years (SD ± 13.9). From a psychopathological perspective, patients scored an average of 11.50 (SD ± 9.9) on the Hamilton Anxiety Rating Scale (HAM-A), while the score reflecting obsessive–compulsive symptoms on the Yale–Brown Obsessive–Compulsive Scale (Y-BOCS) was approximately 10.49 (SD ± 6.9). Table 1 presents the clinical and sociodemographic characteristics of the study sample.

Statistically significant differences emerged between the two groups in the age of onset of the OCD (*p* = 0.083), psychiatric familiarity (*p* = 0.023), HAM-A total score (*p* < 0.0001), and Y-BOCS (*p* < 0.0001). These results are listed in Table 2.

The logistic regression analysis revealed a statistically significant association between the presence of cPTSD and the Y-BOCS total score (*p* < 0.0001), as displayed in Table 3.

## 4. Discussion

### 4.1. Exploring the Interplay between cPTSD and OCD Severity: Insights and Implications

The present study addresses a topic of great interest that is still little debated, probing the intriguing link between cPTSD and the clinical severity of OCD. Our data unveiled a compelling correlation between the presence of cPTSD and a notably exacerbated clinical trajectory of OCD, as evidenced by higher scores on the Hamilton Anxiety Rating Scale (HAM-A) and the Yale–Brown Obsessive–Compulsive Scale (Y-BOCS). These findings are in line with existing literature, reinforcing the notion that cPTSD predisposes individuals to compromised outcomes across a spectrum of psychiatric disorders [19]. Furthermore, our logistic regression analysis has demonstrated a significant association between Y-BOCS scores and the Impact of Event Scale (ITQ), underscoring the profound influence of cPTSD on the augmentation of obsessive–compulsive symptoms. This finding warrants elucidation, as it implies that exposure to traumatic stressors during critical phases of psychological development may catalyze the intensification of affective disturbances and relational challenges. These disturbances, in turn, give rise to disruptions in self-organization, thereby exacerbating obsessive symptoms. This alignment with prior literature substantiates the link between traumatic stressors and the severity of OCD symptomatology [6,58]. The multifaceted consequences of trauma on OCD encompass a spectrum of distressing phenomena, including dissociative symptoms, intrusive thoughts and images, profound anxiety, a sense of helplessness, and heightened vigilance, all of which contribute to the chronicity and severity of the disorder. These manifestations often intertwine, creating a complex web of psychological distress that can significantly impair daily functioning and exacerbate the individual’s overall distress and impairment in quality of life. [59]. Empirical studies have reported that traumas, especially when occurring during childhood, disproportionately impact the obsessive dimension of OCD. Trauma types encompass sexual abuse, adverse living conditions, bullying, and exposure to traumatic events such as bereavement [60,61]. Notably, prolonged exposure to such trauma has been correlated with the emergence of Complex cPTSD, with higher incidence rates among individuals exposed to chronic abuse or neglect [62]. It is pertinent to highlight that interpersonal trauma assumes a pivotal role in the psychopathology of both cPTSD and OCD [48,63,64]. Compared to non-interpersonal trauma, interpersonal trauma has a more profound impact on self-regulation, frequently inducing dissociation [65]. Consistent with contemporary literature, higher rates of childhood trauma correlate positively with increased levels of dissociation, which serves as a mediating factor between trauma exposure and psychiatric symptoms [66].

cPTSD often leads to dissociation, a mental mechanism that can be employed adaptively or maladaptively in response to distressing life experiences [67]. Studies indicate that among individuals with minimal exposure to trauma, dissociation may serve as a normal and even constructive aspect of the mind, possibly linked to typical self-absorption phenomena and creative coping strategies during distressing events [68]. Moreover, among individuals with high levels of repetitive trauma exposure, as seen in cPTSD, dissociation might become pervasive, significantly impacting their overall mental well-being. In such cases, the mind appears to retain a memory of past traumas, with dissociation potentially serving as a primary psychological mechanism organizing the individual’s sense of self and contributing to the development and reinforcement of maladaptive personality traits [69]. This issue plays a pivotal role in exacerbating obsessions and compulsions, while also potentially reinforcing the character rigidity of individuals affected by OCD, leading to isolation and consequently a greater severity of symptoms, as evidenced by our results. This can be attributed to patients with OCD, who have experienced multiple traumatic events, exhibiting a greater propensity for decompensation, a lack of functional and cognitive recovery, and the onset of dissociative symptoms [70,71]. 

### 4.2. Integrating Trauma-Focused Treatments in OCD Management: A Holistic Approach to Healing and Recovery

Cognitive-behavioral and pharmacological interventions and trauma-focused treatments have emerged as crucial components in the comprehensive management of OCD. Recognizing the intricate relationship between trauma and OCD symptomatology, it has become increasingly evident that addressing underlying traumatic experiences is essential for effective treatment outcomes. Trauma-focused therapies aim to directly target the distressing memories, emotions, and physiological responses associated with traumatic events, thereby reducing their impact on OCD symptoms [72]. These interventions provide individuals with the tools and resources needed to process and integrate their traumatic experiences, ultimately facilitating symptom relief and promoting psychological resilience. Mindfulness-based interventions offer individuals a non-judgmental awareness of their thoughts, emotions, and bodily sensations, enabling them to cultivate greater acceptance and self-regulation in the face of distress. Sensorimotor psychotherapy focuses on the interconnection between bodily sensations, emotions, and cognitive processes, allowing individuals to access and process traumatic memories stored in the body [73]. Eye Movement Desensitization and Reprocessing (EMDR) is another evidence-based treatment modality that has shown promise in addressing trauma-related symptoms. By engaging in bilateral stimulation while revisiting traumatic memories, individuals can reprocess these experiences in a safe and controlled manner, leading to a reduction in distress and symptom severity [74]. The integration of trauma-focused approaches within the therapeutic framework of OCD treatment underscores the importance of addressing the root causes of psychological distress and dysfunction. By targeting both the symptoms of OCD and the underlying trauma, clinicians can help individuals achieve meaningful recovery and improved quality of life. As research continues to evolve, it is imperative to further explore the efficacy and mechanisms of trauma-focused treatments in the context of OCD. Continued collaboration between researchers, clinicians, and individuals with lived experience will facilitate the development of tailored interventions that address the diverse needs of those grappling with the intersection of trauma and OCD. Through comprehensive and compassionate care, individuals can find healing and reclaim agency over their mental health journey [75]. Recognizing the complexities introduced by prior prolonged trauma is paramount, as individuals may exhibit resistance to traditional OCD treatment and may resort to alternative maladaptive coping mechanisms to mitigate the distress associated with traumatic memories [76]. An in-depth exploration of the physical, emotional, and psychological turmoil stemming from trauma is essential for tailoring therapeutic approaches, ultimately enhancing the quality of life for patients [16]. Future investigations should focus on elucidating how complex trauma influences an individual’s quality of life and interpersonal functioning. This knowledge can inform psychosocial interventions, including rehabilitation activities aimed at fostering relationships and mitigating social isolation. In our opinion, interventions that focus on the psychosocial aspects are particularly important for patients with OCD comorbid with cPTSD, as the persistence of trauma can exacerbate symptoms, and such interventions would act on various levels, including the interpersonal one. Indeed, merely focusing on the patient’s resilience capacity with OCD might not suffice. Structured psychoeducation sessions involving cohabiting figures, or at least significant ones, would be necessary [77]. All of this would serve to break the continuity of trauma and reduce expressed emotionality, which could re-trigger symptoms while creating an environment and context around the patient conducive to healing. Additionally, it would assist the clinician in identifying triggers that can precipitate and worsen obsessive symptoms. In this regard, psychoeducational interventions involving family members have been developed, but studies are mostly conducted in pediatric and adolescent age groups and do not focus well on trauma [78]. The gold standard should represent a psychoeducational intervention aimed at identifying early signs of decompensation, which in this case are the effects of traumatic events perpetrated over time, and intervening on different levels, such as the following: the family, which in this case should be supportive and collaborate in implementing skills, pharmacological, social, and intervening on the traumatic experience. Psychoeducation modules adapted to traumatic experiences are already present in other disorders, such as bipolar disorder [79]. Therefore, there is still much to be done in this regard. Identifying optimal strategies for enhancing cognitive functioning and managing behavioral disorders holds the promise of improving the well-being of patients and their families. This study serves as an incentive to intensify research efforts, delving into the intricate interplay between complex trauma and the severity of obsessive–compulsive symptoms. The dearth of studies addressing this critical nexus necessitates further exploration, highlighting the urgency of continued research. Additionally, our findings illuminate the earlier onset of cPTSD, likely attributed to prolonged or repeated exposure to interpersonal traumas, such as childhood abuse or domestic violence [74]. Notably, the present study reaffirms the familial component in psychiatric disorders, particularly in cases of early-onset OCD. 

### 4.3. Limitation

There are several caveats to consider when interpreting the findings of this study. Firstly, as is typical in much research within this domain, cPTSD was evaluated retrospectively and through self-report measures. This introduces the possibility of recall bias regarding traumatic events experienced. Nonetheless, research has demonstrated the reliability of self-reporting concerning recurrent traumatic experiences using the ITQ, even in cases where psychopathology diminishes following therapy [76]. Secondly, like other studies exploring the effects of treatment on cPTSD, our investigation focused on an outpatient clinical sample with relatively stable symptomatology. Therefore, the findings may not be generalizable to a more severely affected inpatient population. Thirdly, certain potentially confounding variables, such as the frequency of exposure sessions or the nature of the trauma, were not controlled for. However, the ITQ is adept at discerning cumulative trauma. While it is considered the current standard measure of cPTSD and is widely used in the literature, future research should strive to enhance the reliability of childhood trauma assessment. Encouragingly, multilevel models do not necessitate complete datasets, and missing value analyses indicated no discernible differences in OCD symptom severity or levels of cPTSD between the dropout and completer groups. Moreover, a thorough examination of the accumulated trauma endured by individuals with OCD and the varying impact of distinct forms of trauma should be undertaken. Maercker and colleagues have identified significant distinctions based on the nature of trauma experienced by individuals predisposed to PTSD (such as abduction and sexual assault) versus those prone to cPTSD (childhood abuse and intimate partner violence) [80]. This will serve as the focal point of our forthcoming research. Subsequent investigations should compare different cohorts of individuals with cPTSD, PTSD, and OCD who lack a history of lifetime trauma.

## 5. Conclusions

In conclusion, our study has illuminated the complex relationship between cPTSD and the severity of OCD. Despite recognizing limitations such as sample size and study design, our results emphasize the critical need for a holistic understanding of how trauma influences the course of OCD. It is essential to recognize that trauma can significantly shape the development and manifestation of OCD symptoms. This understanding is crucial for developing interventions that address the multifaceted effects of trauma and its profound impact on individuals’ lives.

Moving forward, future research efforts should build upon these findings to create a comprehensive framework aimed at improving the well-being of individuals grappling with the intersection of complex trauma and obsessive–compulsive challenges. By delving deeper into these dynamics, researchers and clinicians can better tailor interventions to address the unique needs of individuals with comorbid cPTSD and OCD, ultimately enhancing their quality of life and promoting recovery.

## Figures and Tables

**Table 1:** Sociodemographic characteristics of the sample (N = 107).

Variable	N or Mean	% or SD
Male	54	50.5
Diploma	84	78.5
Presence of psychiatric familiarity	74	69.2
Presence pf cPTSD	50	46.7
Age	45.75	13,902
HAM-A total score	11.50	9999
Y-BOCS total score	10.49	6931

HAM-A: Hamilton Anxiety Rating Scale; Y-BOCS: Yale–Brown Obsessive–Compulsive Scale; cPTSD: complex posttraumatic disorder; N: total number; SD: standard deviation; %: percentage.

**Table 2:** Analysis of variance between the group with and without cPTSD.

Variable	df	F-Value	*p*-Value
Age of OCD onset	1	3.073	**0.083**
Familiarity with other disorders	1	0.065	0.799
Psychiatric family history	1	5.333	**0.023**
HAM-A total score	1	64.803	**<0.0001**
Y-BOCS total score	1	38.462	**<0.0001**

HAM-A: Hamilton Anxiety Rating Scale; Y-BOCS: Yale–Brown Obsessive–Compulsive Scale; cPTSD: complex posttraumatic disorder; df: degrees of freedom. Bold *p*-values indicate statistical significance.

**Table 3:** Logistic regression beetween complex Post Traumatic Stress Disorder and Y-BOCS total score.

Variable	Beta	SD	Wald Statistics	df	*p*-Value
Y-BOCS_TOT	0.202	0.043	22.111	1	<0.0001
Intercept	−4.759	1.028	21.453	1	<0.0001

Y-BOCS: Yale–Brown Obsessive–Compulsive Scale.

## Data Availability

The data supporting the conclusions of this study can be obtained from the corresponding author upon a reasonable request.
